# External inspection approaches and involvement of stakeholders’ views in inspection following serious incidents - a qualitative mixed methods study from the perspectives of regulatory inspectors

**DOI:** 10.1186/s12913-024-10714-9

**Published:** 2024-03-06

**Authors:** Sina Furnes Øyri, Siri Wiig, Janet E. Anderson, Inger Johanne Bergerød

**Affiliations:** 1 SHARE – Centre for Resilience in Healthcare, Department of Quality and Health Technology, Faculty of Health Sciences, University of Stavanger, Stavanger, Norway; 2 Stavanger University Hospital, Stavanger, Norway; 3 Department of Anaesthesiology and Perioperative Medicine, Faculty of Medicine, Nursing and Health Sciences, Monash University, Melbourne, Australia

**Keywords:** External inspection, Stakeholder involvement, Serious incidents, Formal objectives, Public expectations, Communication and interaction

## Abstract

**Objective:**

The objective was to gain knowledge about how external inspections following serious incidents are played out in a Norwegian hospital context from the perspective of the inspectors, and whether stakeholders’ views are involved in the inspection.

**Methods:**

Based on a qualitative mixed methods design, 10 government bureaucrats and inspectors situated at the National Board of Health Supervision and three County Governors in Norway, were strategically recruited, and individual semi-structured interviews were conducted. Key official government documents were selected, collected, and thematically analyzed along with the interview data.

**Results:**

Our findings overall demonstrate two overarching themes: Theme (1) Perspectives on different external inspection approaches of responding and involving stakeholders in external inspection following serious incidents, Theme (2) Inspectors’ internal work practices versus external expectations. Documents and all participants reported a development towards new approaches in external inspection, with more policies and regulatory attention to sensible involvement of stakeholders. Involvement and interaction with patients and informal caregivers could potentially inform the case complexity and the inspector’s decision-making process. However, stakeholder involvement was sometimes complex and challenging due to e.g., difficult communication and interaction with patients and/or informal caregivers, due to resource demands and/or the inspector’s lack of experience and/or relevant competence, different perceptions of the principle of sound professional practice, quality, and safety. The inspectors considered balancing the formal objectives and expectations, with the expectations of the public and different stakeholders (i.e. hospitals, patients and/or informal caregivers) a challenging part of their job. This balance was seen as an important part of the continuous development of ensuring public trust and legitimacy in external inspection processes.

**Conclusions and implications:**

Our study suggests that the regulatory system of external inspection and its available approaches of responding to a serious incident in the Norwegian setting is currently not designed to accommodate the complexity of needs from stakeholders at the levels of hospital organizations, patients, and informal caregivers altogether. Further studies should direct attention to how the wider system of accountability structures may support the internal work practices in the regulatory system, to better algin its formal objectives with expectations of the public.

**Supplementary Information:**

The online version contains supplementary material available at 10.1186/s12913-024-10714-9.

## Introduction

Norway has a universal public healthcare system entailing different accountability structures as key strategies to ensuring and improving quality and safety [1]. External inspection is one of these accountability structures. Along with accreditation, external inspection is an internationally wide-spread external evaluation method used to assess the quality of care given to patients [2, 3]. The aim is to ensure that healthcare providers adequately implement, maintain, and improve quality and safety [2, 3]. In a broader sense, external inspection contributes to enhancing healthcare providers’ processes of risk management [4]. Previous research has explored how regulatory inspectors approach healthcare organizations and in what ways external inspection potentially could impact quality improvement [5–13]. There is however conflicting evidence about how and if regulatory activities, including external inspection, is designed in ways that factor in the complexity, uncertainty, and variation in healthcare, and whether it is performed with the sufficient knowledge, expertise, and skill set in stakeholder involvement [9, 11, 13–18]. In an international context, external evaluation of quality and safety, and national policy strategies, have different organizational structures, including differences in regulatory implementation and enactment processes [2, 4]. The Norwegian system of regulatory, external inspection specifically is a mandatory mechanism designed to control quality and safety systems, implemented by healthcare providers [4]. The system is based on principles of internal control of structure and process, where attention to management responsibilities and system-performance is given over attention to individual performance [4] (please see Table 1). In contrast, the US system of external evaluation rests on voluntary, non-statutory accreditation, individual liability in cases of medical error countered by a system of torts; insurance, with management-oriented evaluation of structure and process by a compliance-based focus [4]. Although each country has its own characteristics, the Norwegian regulatory context featuring of a state regulated and mandatory system design for external evaluation, bears resemblance to other European countries such as the Netherlands [2, 4, 5].

In recent years, the WHO and national governments across the globe seem to have become more interested in the idea of gaining contributions from different stakeholders, to improve quality and safety [3, 19–21]. Studies have encouraged a more extensive and structured approach to stakeholder involvement in risk and safety regulation and external inspection, for instance by influencing the regulatory design process [22–30] and by collecting the perspectives of patients and families following serious incidents [31, 24, 25, 32–34]. A recent review offers indications of an ongoing change of course to involving patients and informal caregivers directly in incident-based inspections, and it suggests that this shift is more evident in external inspection culture and methods than in processes of accreditation [4]. Except from a couple of reports and one study targeting next of kin involvement following regulatory inspection of serious incidents, knowledge is scarce in the Norwegian context about how stakeholder involvement unfolds in regulatory inspection, including what the implications might be [24, 25, 35, 36]. The study presented in this paper is therefore innovative: it reports mixed-methods qualitative findings of regulatory inspectors’ perspectives on their approaches of responding to an incident, and how their work concerning serious incidents involves and is informed by stakeholders. Stakeholder involvement was in this study defined as healthcare related processes or decisions concerning external inspection which may become informed by multiple stakeholders from different system levels such as: hospitals and healthcare professionals, patients, and informal caregivers [37].

The Norwegian System of Regulating Quality and Safety in Healthcare.

The Norwegian Board of Health Supervision (NBHS) and 11 regional County Governors perform system audits (systematically planned inspection), or incident-based inspection to follow up serious incidents reported directly to the NBHS [38, 39]. The legal framework of external inspection is comprehensive, including objectives, requirements and processes found in the act relating to external inspection in the healthcare services [40] and in the act relating to the specialized healthcare services [41]. The Ministry of Health and Care Services (MHCS) provides the NBHS and County Governors with additional regulations, policies, and guidelines for the regulatory activities expected to be carried out by these bodies, including policies for stakeholder involvement. The principle of sound professional practice informs the inspector’s assessment of whether adequate quality and safety in health care has been provided or not [42]. Hospitals and health professionals are required to report serious incidents, and the regulatory framework directs the enforcement of this duty to the managerial level, and not towards individual health professionals [40–42]. Please see Table 1 for definitions and key facts about the Norwegian system of regulating quality and safety in healthcare.

**Table 1:** Definitions and key facts about the Norwegian system of regulating quality and safety in healthcare

• The regulatory framework sets out three generic criteria for incidents being considered “serious”, and thus require reporting the national reporting system [40–42]. These criteria are: (1) The patient dies or is subject to severe harm, (2) which is considered a result from the treatment given (or lack of treatment), (3) and where the outcome is unexpected due to expected risk.
• In addition to the hospitals’ and health professionals’ obligation to report, the public, patients, and informal caregivers have the *right to* report [43]. Reports are supposed to be registered through the national reporting system and government-based web site called “melde.no”.
• Patient injuries registered for 2022 in Norwegian hospital settings, measured by Global Trigger Tool, were shown to have a slight reduction, with a patient injury occurring in 12,6% of hospital stays in 2022 against 12,8% in 2021 [44].
• In 2021, a total of 4473 cases of serious incidents were registered by the County Governors. 856 out of 2241 cases were assessed, with one or more violations of legislation appointed in 38% of the cases. 225 cases were forwarded to the Norwegian Board of Health Supervision for potential administrative reaction against individual health professionals. 104 planned inspections in the specialized healthcare services were conducted [45].
• Hospital internal risk management and quality improvement efforts should be based on the Quality Improvement Regulation (QIR), a regulatory, national framework for the managerial role and managerial responsibilities in relation to quality and safety enhancing work [46].
• Evidence-based guidelines are developed and implemented at local and/or regional organizational system levels. National-based guidelines are developed by the Norwegian Directorate of Health to support the services in their application of state-of-the-art knowledge and to facilitate consistency of the services offered across the country [47].
• Registries and national quality indicators are administered by the local and regional health trusts and the Norwegian Directorate of Health, and the indicators are expected to be applied by the hospitals in their efforts to improving quality [48].
• Fines and revocation are available regulatory responses being administered and issued by the Norwegian Board of Health Supervision [40]. Fines are targeting the organizational level, while revocations are targeting individual health professionals [40].

### Objective and research question

The objective was to gain knowledge about how external inspections following serious incidents are played out in a Norwegian hospital context, from the perspective of the inspectors, and whether stakeholders’ views are involved in the inspection.

The main research question was: how do different inspection approaches facilitate inspectors to involve stakeholders’ views in the inspection following serious incidents?

## Methods

### Study design and setting

This is a qualitative mixed-methods study [49] combining the perspectives of regulatory, external inspectors at the government level with expectations of stakeholder involvement identified in governmental documents. According to Morse and Niehaus [49, pp. 17–18], a study is considered mixed method if it consists of a core component and a supplemental component, published in the same scientific paper. In our study this is illustrated by combining qualitative methods of interview data (the core component), supplied by documents, altogether published in this present paper. This methodological strategy was chosen as it allowed us to explore different aspects of the same phenomenon played out in a Norwegian hospital context: expectations formed in official governmental documents, and perspectives given by the inspectors.

#### Participant recruitment & characteristics

10 participants were recruited by strategic sampling and approached by e-mail [50, 51]. The NBHS participants were contacted based on identification done by a well-experienced informant, who provided us with relevant participant names and contact information. Participants at the County Governor level were identified based on requests sent by e-mail to the relevant County Medical Officer or unit manager for the relevant specialized healthcare service. The researchers aimed at participants holding in-depth insights into hospital external inspection of serious incidents. The participants were seven women and three men. Five of them were medical doctors and five lawyers (one being a registered nurse). Two participants were situated at the national level of NBHS whilst eight participants were located at three County Governors.

### Data collection

Data was collected by individual interviews and relevant official, governmental documents such as acts, regulations, guidelines, and reports concerning external inspection (see Table 2). The interviews were conducted in person at the workplace of the participant, by researcher SFØ and researcher IJB between March 2022 and June 2022. Interviews lasted approximately 1 h and were conducted in Norwegian. The researchers used a semi-structured interview guide (see supplementary file) covering the topics: the role of regulatory inspectors, methods, self-assessment template, onsite inspection, guidelines and support material for internal work practices, involvement such as interaction and dialogue with hospital organizations, health professionals, patients, informal caregivers, media attention, and areas of possible future development in external inspection. This strategy allowed the researchers to ask relevant follow-up questions. All interviews were recorded and subsequently transcribed by an external consultant. The documents were identified and selected based on researcher SFØ’s pre-existing familiarity with the subject of external inspection [13]. Hand searches were thus required for this part of the data collection. The document referred to as “Guidelines for inspection conducted by the County Governor level- Appendix 2” [52] was however forwarded to the researchers, after the interview was done.

**Table 2:** Documentary evidence identified, included and analyzed

• Patient- and informal caregivers’ perspectives following serious incidents [35].
• The Act of 2 July 1999 No. 64 relating to Health Personnel (the Health Personnel Act) [42].
• The Act of 2 July 1999 No. 61 relating to the specialized healthcare services (the Specialized Healthcare Services Act) [41].
• The Act of 15 December 2017 No. 107 relating to governmental supervision with the healthcare services (The Health Supervision Act) [40].
• Guidelines for inspection conducted by the County Governor level- Appendix 2 [52].
• Recommendations related to stakeholder involvement in external inspection [53].
• Information to managers and health professionals regarding onsite inspection [54].
• Annual Report 2021 Norwegian Board of Health Supervision [39].
• Onsite inspection as method following reports of serious incidents [55].
• Guidelines - reports delegated to the County Governors from the NBHS [56].
• Response letter from the NBHS to the Consultative Committee responsible for assessing the incident reporting regime in Norway [57].
• Reminder: letter from the NBHS to the health care trusts and municipalities [58].
• Additional external inspection follow-up following reports of serious incidents [59].
• External inspection follow-up of violations [60].
• Follow up by the organization and feedback to the County Governor [61].
• Priorities and assessment regarding external inspection [62]
• Reception and clarification - Guidelines for the County Governor proceedings of incidents [63]
• Delegation to the County Governor of notifications related to serious incidents [64]
• Transfer to organizations/health professionals [65].

### Analysis

Interview data as the primary data source, and supplementary documents were analyzed by thematic analysis [51]. The inductive approach by the application of thematic analytical principles has a data driven, bottom-up way of analysis [51]. Hence, and as described by Braun & Clarke (51 p. 178), the approach does not require a theoretical framework or ontological or epistemological ideas.

This paper presents the findings from the thematic analysis altogether. The analysis was done inductively using codes as building blocks for overarching themes. Interview transcripts were analyzed by researcher SFØ and researcher IJB in four steps: (1) open reading process of interview transcripts (2) identifying meaning units, key words, and questions for reflection (3) forming and discussing codes, and (4) refining overarching themes. Document analysis data was used to contextualize the interview data and provide explanations for governmental expectations of the inspectors’ methods and approaches of responding to an incident, and to understand the expectations to stakeholder involvement practices in inspection [66]. The process involved thorough reading of all documents, and identification of relevant passages of text related to key words such as onsite inspection, self-assessment, objective/aim, involvement, information [66]. The researchers SFØ and IJB had several meetings discussing and refining patterns of meaning, codes, and themes.

### Trustworthiness

As part of the process of ensuring relevance and trustworthiness, the research protocol and interview guide were presented to fellow researchers and members of a panel of different stakeholders situated at various levels of the healthcare system (please see *Patient and Public Involvement Statement*.

below). The interview transcripts displayed information rich participant responses, covering a wide collection of inspection related topics. Our efforts to ensure trustworthiness throughout the process of analysis and presentation of findings were focused on providing as much methodological context as possible, and by tight analytical collaboration between researcher SFØ and researcher IJB [67].

### Patient and public involvement statement

The study presented here is part of a larger research project on stakeholder involvement in external inspection and hospital internal assessment of serious incidents. The main project plan was presented to SHARE– Centre for Resilience in Healthcare’ Stakeholder Panel, in an early phase, and feedback was provided by designated members of the panel. We also involved a highly experienced researcher, who provided valuable insights and feedback to the project plan and interview guide (please see Acknowledgement).

## Results

The thematic analysis resulted in two overarching themes. Each overarching theme was accompanied by codes, presented in bullet points for each theme.

Theme 1. perspectives on different external inspection approaches of responding and involving stakeholders in external inspection following serious incidents.

- Five key approaches of responding, used in incident-based inspection with intention to increase stakeholder involvement.
- Frequent hospital application of self-assessment templates in inspections.
- Infrequent on-site inspection in hospital settings.

Theme 2. Inspectors’ Internal Work Practices Versus External Expectations.

- The formal objectives of external inspection encountering patients’ and informal caregivers’ understanding and perception of quality and safety.
- Sound professional practice principles encountering patients’ and informal caregivers’ understanding and perception of quality and safety.
- Governmental delegation of responsibility for incident-based inspection; Resource Demands.

In the following we present interview and document findings altogether by summary of each overarching theme and codes, including participant quotations. In Table 3 below, we provide example citations retrieved from some of the documents for each of the two themes and their belonging codes.

**Table 3:** Theme 1 and 2, codes, and examples of document citations

Code	Document Citations
**THEME 1 Perspectives on different External Inspection Approaches of Responding and Involving stakeholders in External Inspection following Serious Incidents**	
Five key approaches of responding, used in incident-based inspection with intention to increase stakeholder involvement	“The County Governor must assess which procedures are the most appropriate to get the necessary progress in the (internal) improvement work, and what will be most expedient in each individual case. How extensive and close the follow-up should be depends on a specific assessment of the content of the case and the conditions in the (healthcare) organization”. *NBHS guidelines to the County Governors ref. 12.06.2023* [60].
Frequent hospital application of self-assessment templates in inspections	“The (healthcare) organization is responsible for patient safety and quality improvement work. By application of this approach, we perform inspection of whether the organization’s follow-up processes are in accordance with the Quality Improvement Regulations”. *NBHS guidelines to the County Governors ref. 12.06.2023* [60].
Infrequent on-site inspection in hospital settings	“When the NBHS receives a notification (…), it should conduct on-site inspection as soon as possible, if this is necessary in order to sufficiently inform the case”. *The Health Supervision Act, § 6* [40]. “When the department conducts on-site inspection, we always offer a meeting involving the patient/user and/or informal caregivers. The final report from onsite inspection is always returned to the patient/user and/or informal caregivers”. *NBHS Annual Report 2021* [39].
**THEME 2 Inspectors’ Internal Work Practices Versus External Expectations**	
The objectives of formal inspection encountering patients’ and informal caregivers’ understanding and perception of quality and safety	“The purpose of the Act is to establish government inspection which contributes to strengthening the safety and quality of the healthcare services and the public’s trust in healthcare professionals and the healthcare services”. The Health Supervision Act, § 1 [40]. “The experiences of users and informal caregivers from encounters with the (healthcare) services are important information when the Norwegian Board of Health Supervision prioritizes the use of external inspection resources. The experiences contribute to our inspection becoming relevant and to improving the quality and safety of the services. Involvement of users and informal caregivers is an important contribution to strengthening the public trust in the services”. NBHS Annual Report 2021 [39].
Sound professional practice principles encountering patients’ and informal caregivers’ understanding and perception of quality and safety	“Healthcare services offered or given in accordance with this Act should be (in line with the principle of) sound professional practice”. MHCS, 1999; the Specialized Healthcare Services Act, § 2–2 [41]. “Those who request the external inspection authority’s assessment of an incident may often have an expectation that we should conclude whether the health care provided was sound; adequate. The individual’s wishes for supervisory follow-up will in some cases conflict with the need we have to direct our efforts towards conditions that will lead to increased quality and safety for more people (than the individual). This must be clearly communicated, and we must explain why we prioritize the way we do. At the same time, we need to take care of the person who approached us”. NBHS guidelines to the County Governors ref. 12.12.2022 [62].
Governmental delegation of responsibility for incident-based inspection; Resource demands	“The County Governor is given the authority to supervise the healthcare services and is directly subordinate to the Norwegian Board of Health Supervision”. *The Health Supervision Act, § 3* [3, 40]. “If the early case proceedings indicate that the case should be transferred to the County Governor for further process, we leave the County Governor with the overall contact with the patient/user/informal caregiver(s)”. *NBHS Annual Report 2021* [39].

### Theme 1: Perspectives on different External Inspection Approaches of Responding and Involving Stakeholders in External Inspection following Serious Incidents

#### Five key approaches of responding, used in incident-based inspection with intention to increase stakeholder involvement

Overall, documents demonstrated that incidents reported through the national reporting system, were subject to a multi-element decision-making process [63]. Findings demonstrated application of five different approaches for responding to an incident, by external inspectors. These were described in the document “Guidelines for inspection conducted by the County Governor level” [52] and supported by the participants. The approaches were related to:

1. Guidance: Termination and guidance offered (the incident reported is not processed any further).
2. Internal handling: The incident reported is forwarded to the hospital for internal handling solely.
3. Self-assessment: The hospital is provided with a template and handles the follow-up of the incident internally by doing a written self-assessment as instructed in the template. The template is returned to the NBHS or the County Governor, which gives feedback to the hospital.
4. Meeting: A meeting is held between the NBHS, and the hospital management team, or between the County Governor, hospital, and the patient, and/or informal caregivers.
5. On-site inspection: The NBHS or the County Governor explores the incident by incident-based inspection, and/or on-site inspection, and makes a final decision about the outcome of the case.

These five approaches may be supplied or combined with for instance collection of statistics, random sampling of patient journals, or the inspection body may encourage the hospital to seek external assistance in its quality improvement work or offer guidance on how to follow up healthcare professionals [63]. Some participants gave accounts of a certain flexibility among the range and combinations of different external inspection approaches, for instance that the inspector could make a phone call to the patient or informal caregiver to clarify or supply case matters with additional information. Few of the participants did however reveal their perspectives related to how they supplied or combined elements from all five inspection approaches available. Participants primarily spoke about the inspection approaches of self-assessment and onsite inspection.

“We have received signals from the Norwegian Directorate of Health that they think it would have been nice if we had continued to work on expanding the toolbox a little, because different types of tools are probably suitable in different contexts. Hence, there is no reason, (…) to only have one method or approach available. It might be a good idea to have a small “bouquet” of different methods that we can use”. *NBHS, interview 1.*

Participants described how incidents were discussed in what they referred to as a “start-up meeting”, where decisions were made on how to proceed. Documents showed that an incident reported to the NBHS may become terminated and not proceeded further based on three arguments: (1) due to the incident being out of regulatory scope, (2) due to the incident being sufficiently informed and with no deviation suspected, (3) or due to the incident needing more investigation before a final assessment can be reached [52]. Severity of the incident, complexity and potential risk for recurrence should be considered and form the decision on how to proceed further [64].

“(…) when we do the sorting of the incidents reported to us, we decide on how to- or which method to choose to get the most out of it. For example, what is appropriate for us to investigate, and how can we best help the services to improve”. *County Governor 1, interview 4.*

If the NBHS decides to move forward with the serious incident reported, the NBHS either chooses to conduct onsite inspection or do “other external inspection follow-up”, as described in the Guidelines issued [52, 56]. The latter includes all the other types of methods mentioned among the overall five inspection approaches and can either be applied and performed by the NBHS, or by the County Governors. These approaches are however frequently applied by the County Governors, because the NBHS forwards most of these cases to the County Governors [61, 64]. The choice of follow-up from the County Governor’s part is based on the decision letter issued by the NBHS, which concludes with violation of legislation, and confirms the need to proceed with “other external inspection follow up” [61]. The autonomy is however substantial as the County Governors may “consider which strategies that are most applicable to obtain quality improvement” [65].

All participants reported development towards greater variety of external inspection approaches, and a stronger incentive to involve, inform and apply information from patients and/or informal caregivers. These accounts were reflected in new policy documents developed at the national government level. In its Annual Report, the NBHS states that the leading principle is to retrieve information from patients and informal caregivers, in all types of serious incidents reported. The NBHS considers it particularly important if a case is terminated/not further processed [39].

Since 2014, the NBHS have issued several documents, for instance recommendations for sound stakeholder involvement, the “Patient- and informal caregivers’ perspectives following serious incidents” document as well as established a user panel aiming to ensure that experiences from users, patients and informal caregivers are taken into consideration in activities related to external inspection [35, 53]. Adding to this is the statement issued by the NBHS, emphasizing its expectations towards the services: to ensure follow-up routines and processes of involving patients and informal caregivers, a “highly prioritized task” [58].

Our participants reported that communication and interaction with patients and/or informal caregivers could contribute positively to revealing the complexity of the incident as well as informing the decision-making processes of the inspectors. However, and despite policies and guidelines pointing to the importance of stakeholder involvement, participants pointed that communication and interaction with patients and/or informal caregivers could be a challenging part of their job. This was described as due to several elements, such as time management and resources, lack of experience and/or relevant competence among the inspectors, the patient and/or informal caregiver not wanting to be part of the inspection process, disagreement between inspectors and the hospitals and patient/informal caregivers. These aspects could intensify the conflict between the different stakeholders, some participants argued.

#### Frequent hospital application of self-assessment templates in inspection and infrequent on-site inspections in hospital settings

Participants described hospital self-assessment as the inspection approach they selected and applied most frequently. Benefits were described to be increased sense of ownership and responsibility at the hospital organizations.

“The fact that we have tried to work more along the lines of asking what the hospital internally investigates the incidents, and we have started to test the method of self-assessment where the idea is that the inspectors their process. We get a lot of positive feedback from that, simply because it increases ownership and responsibility within the hospital organization”. *NBHS, interview 1.*

As demonstrated in the “Guidelines for inspection conducted by the County Governor level” document, the self-assessment report was seen as beneficial to the adversarial principle because the report may be forwarded to the patient or informal caregiver for comments and feedback [52, 56]. According to the NBHS, this process could in turn encourage the hospitals to set up a dialogue with the informal caregivers [65].

“Regarding self-assessment, we think that it’s a document we can return (based on the adversarial principle) to obtain the views of informal caregivers. However, it is even better- we do have examples of it- the hospitals initiate dialogue and get their aspects included into the self-assessment”. *County Governor 2 interview 7.*

Based on our data, it was however not possible to confirm if the self-assessment template included standardized question(s) about involving the patient and/or informal caregivers. The general impression from the documentary evidence and interviews was nevertheless that the template often asked for information about how informal caregivers and the affected health professionals were taken care of in the aftermath of the serious incident [52, 56, 61, 65].

“We often get the cases we select for the approach of self-assessment in return from the hospital because the patient or the informal caregivers inform us that they already have attended meetings at the hospital, and they do not trust the hospital. Then we will have to request a “full-scale version” of inspection (….) which is a dilemma”. *County Governor 2, interview 8.*

Despite the overall findings indicating positive and valuable aspects of applying self-assessment as a means of involving hospitals more thoroughly in the external inspection processes and internal improvement process, some of the participants mentioned drawbacks with the approach. They pointed out that self-assessment primarily facilitated written communication between the regulatory inspectors and the hospital. This approach could potentially conceal important issues or internal aspects, participants argued.

Onsite inspections were described by our participants as less frequently used compared to other approaches. Some participants however pointed out that having a close dialogue with the hospital was more important than the sole use of onsite inspection. Some emphasized that they wished they used onsite inspection more frequently despite the approach being demanding resource wise. According to the Act relating to external inspection for instance, the NBHS should conduct an onsite inspection following a report of a serious incident, “as soon as possible” [40]. The requirement is however reserved for serious incidents where onsite inspection is necessary to get the case “sufficiently disclosed” [40]. Documents showed that if an onsite inspection is to be carried out, the NBHS offers the patient or the informal caregiver(s) a conversation, where their experiences, observations and reflections are seen as important sources of information [55]. During an onsite inspection, the patient or informal caregiver has the right to- and gets forwarded copies of relevant case related documents [5]. Suspicion of poor treatment of the patient or the informal caregivers following a serious incident, was considered one of the key arguments in the NBHS’s decision to do an onsite inspection [55]. Documents however also displayed that the County Governor may choose to do onsite inspection as part of its following up process, to reveal whether the hospital has implemented sufficient changes and/or achieved improvement [65].

“If a serious incident is reported, I would argue that onsite inspection is a much swifter method to disclose what happened. And- if it is a very serious incident where the risk is still ongoing, I believe (onsite inspection) is the best method. It requires enough resources– it is a matter of capacity…it requires a lot of (resource) capacities for as long as the onsite inspection lasts (…)”. *County Governor 2, interview 6.*

Overall, the external inspection approaches demonstrated in our findings, displayed different ways of involving stakeholders, at varying system levels. Onsite inspection was shown in formal documents to have incentives for involvement of patients and informal caregivers, related to their right to be notified prior, during and after the inspection. The option of providing the patient and/or informal caregivers with the draft of the report, and their right to comment on findings, as well as the option of getting access to the proceedings, indicate how perspectives from patients and/or informal caregivers are considered valuable to external inspection of serious incidents [39]. Participants reported that the recent introduction of self-assessment as an inspection approach signaled stronger emphasis on involvement and served as motivation for the hospitals to contribute during the inspection process. It was also described to play an important part of the internal improvement processes in the hospitals undergoing inspection.

### Theme 2: Inspectors’ Internal Work Practices versus External Expectations

#### Formal objectives of external inspection and sound professional practice principles encountering patients’ and informal caregivers’ understanding and perception of quality and safety

Documents showed that the primary objective with external inspection is to enhance patient safety and promote improvements and learning [40]. Participants however expressed some doubt about how their work really contributed to safer and higher quality of care. Corroborated by both participants and documents, findings showed that external inspection is based on a joint medical and legal assessment of sound professional practice [40, 41]. Thus, the inspectors’ work practices were in general lead by the principle of sound professional practice and the considerations about whether adequate care was given patients in the incidents reported. The formal objectives of external inspection were however described to be encountered by the patients’ and informal caregivers’ understanding and perception of the principle of sound professional practice. Some of the inspectors argued that patients and informal caregivers sometimes had different views on what the incident was caused by and interpreted the principle of sound professional practice and considered adequate care differently than the inspectors’ medical-legal assessment did. The process of balancing medical aspects with a legal assessment was described as a team-effort, with a mix of different competencies within a team of inspectors. Thus, most participants considered their internal work practices to be part of a trade-off between different perspectives of quality and adequate care. The objectives of external inspection work were subject to continuous development, they argued.

“The outcome of the case, or the seriousness of the incident, does not necessarily mean that there have been deviations or that it was not in line with sound professional practice. They (patient; informal caregivers) do not understand that- it is difficult to explain it (to them)”. *County Governor 1, interview 3.*

Documentary findings showed how the government suggested inspectors to triangulate different sources of knowledge from patients, informal caregivers, healthcare professionals and managers because triangulation may provide a more complete picture of what happened [53]. In turn, it may provide the inspectors with solid ground for their subsequent assessment concerning sound professional practice and adequate health care [53]. Participants in this study however described it to be a challenging part of their work, balancing the formal objectives with the emotional experience and trust of the patient and/or informal caregivers. Participants described how external expectations formed in government policies and regulations contrasted with the expectations from the public in general, and from patients and informal caregivers specifically. This gap was described as somewhat contradictory, with the strong set of patient and informal caregivers’ rights on one hand and external inspection resources available on the other. Participants saw this gap as a fundamental conflict in the current external inspection system. Another part of this gap was described as the governmental expectation of applying a system-perspective to external inspection of serious incidents, and the perspectives of individual-oriented proceedings of incidents reported where patients and/or informal caregivers sporadically sought to blame individual healthcare professionals. Expectations of accountability and responsibility from the perspectives of the patient and/or his or her informal caregivers, were thus sometimes perceived differently than what the formal joint medical, legal assessment allowed regulatory inspectors to determine.

“I have had quite a few conversations with people, for instance one prior to the weekend, where I spoke to a patient who had complained several times about the same situation, but he got really annoyed because he thought it was strange that we had not concluded that his case was a regulatory violation. Somehow, he did not understand how we could call it sound professional practice because he had contracted cancer several years ago of which the hospital had given him the wrong diagnosis, they had not called it cancer, but it turned out later that it was cancer. He still does not understand how we can call it sound professional practice”. *County Governor 1, interview 4.*

The response letter from the NBHS to the Consultative Committee responsible for assessing the incident reporting regime in Norway argued that many patients and informal caregivers were disappointed if their case was terminated and not followed through [57]. Participants expressed frustration about these issues and reported that the different perceptions of what external inspection could contribute to in terms of enhancing quality and safety, challenged their internal work practices. It moreover held to account the public trust issue described. The participants explained how public perception of sound professional practice, shaped for instance by media attention, could exceed both the formal objective of external inspection as system-level quality improvement and learning, as well as sound professional practice principles. Interaction and communication between inspectors, hospitals and health professionals and patients and informal caregivers became extra challenging in those types of cases, participants argued.

#### Governmental delegation of responsibility for incident-based inspection; resource demands

According to the Annual Reports by the NBHS, the County Governors have in recent years been provided with policies and guidelines of more autonomy and responsibility to decide and prioritize inspection (with regards to risk and relevance) [39, 45]. The annual reports confirm that if an incident is delegated to the County Governor in an early phase of the proceedings, the NBHS leaves all encounters and contact with the patient and/or the informal caregivers to the County Governor [39, 45]. Our participants however, indicated challenging issues with governmental delegation of responsibility for incident-based inspection. The inspectors at the County Governor level believed that the NBHS more often delegated cases of serious incidents to them now, than previously. Due to available internal resources and an overall bigger case-volume being reported, this was portrayed as a challenge. An overall increase in case volume for both the NBHS and the County Governors was raised as an issue that effected the internal capacity in the government bodies [45]. Document data from the NBHS moreover showed that of 763 serious incidents reported from the specialized healthcare services, 576 of the incidents were delegated to the County Governors [39]. 132 incidents reported were terminated and not processed further, six on-site inspections were conducted, 20 incidents were pursued as “other external inspection follow-up” and 29 of the incidents reported were out of scope [39]. These numbers indicate that a substantial part of the serious incidents reported become delegated to the County Governors for further follow up and decision-making. This aligns with the perspectives of our participants. Considering the issue of governmental delegation of responsibility for incident-based inspection, the inspectors stressed the efficient use of resources as an important future interest.

“(…) Due to the fact that the number of incident reports has increased, there is actually a very large part of the resources that go into the initial handling of the reports (….) One outcome may be that the report is forwarded to the County Governor for assessment. It is not like we are instructing them on what to with the case, (…) they consider how to handle it further. However, the delegation process is a bit demanding between us and the County Governors”. *NBHS, interview 1.*

In their annual report, the NBHS acknowledged that the internal work practices at the County Governor level needed coordination and development, to make the use of existing resources “optimal” [39]. Our participants on the other hand, indicated fundamental issues with how external stakeholders perceived and confused the different government bodies involved in external inspection. Some pointed to a potential need to actively clarify the responsibilities and collaboration between the NBHS and the Country Governors, to the healthcare services. This could contribute to an increased understanding of the roles and objectives, some participants argued. Moreover, they emphasized how external inspection in the future should focus more on specific topics of quality and safety and specific regulatees, and not add to the workload of the healthcare services. This could potentially contribute to more relevant and constructive external inspection and increase the legitimacy of the system.

“I think that we sort the cases more diligently than the NBHS expected us to do, because looking into every case is not possible, and we need to be strict in setting our priorities. (…) Even when we are strict, we have a very long case processing time”. *County Governor 1, interview 1.*

## Discussion

### Principal findings

We have explored different approaches of external inspection related to stakeholder involvement in regulatory follow up of serious incidents- as seen from the perspectives of 10 inspectors, supported by document analysis. Principal findings indicated that new approaches of responding have developed and applied to the inspectors’ repertoire of internal work practices. Self-assessment and onsite inspection were found to be most frequently discussed and applied, where the reliance on hospital self-assessment was reported to having benefits but also potential downsides. Reports were given of a development towards more policy and regulatory attention to and sensible involvement of stakeholders such as hospitals, patients and/or informal caregivers. Some of these approaches were perceived to have a stronger incentive for involving hospital organizations, patients and/or informal caregivers than others. Participants agreed that although involvement could entail challenging interaction with stakeholders, it could contribute to positively informing the case complexity and the inspectors’ decision-making processes. The complexity of balancing all perspectives, from patients, informal caregivers, the public in general, health professionals and hospitals, including the high emotional component and the possibility of patient/families being dissatisfied with the inspection approaches and processes, were considered a challenging part of the work led by the inspectors. The increase in incidents reported raises questions about how to resource this work moving forward. Acknowledging the different perceptions of external inspection and clarifying the responsibilities, collaboration, and resources between the NBHS and the Country Governor, were reported key to the development of future external inspection.

### Developing the scope of external inspection and clarifying public expectations

Research has shown that perspectives from patients and informal caregivers, have increasingly become a “key principle” in external evaluation of the healthcare services [4, 32, 33, 68]. However, recent research findings indicate that there is a difference in stakeholder involvement depending on the type of external evaluation system. In accreditation systems, patients and informal caregivers may be less involved compared to inspection designed systems [4]. Knowledge about how different system designs promote stakeholder involvement following serious incidents, is still missing. These indications were corroborated by our findings showing reports of extensive government attention to stakeholder views and the approaches applied by the inspectors. Adding to this is the increasing emphasis on management and the internal work processes of managers in healthcare organizations, set to improve quality and safety, which in turn may have implications for external inspection [69–72]. For instance, in the Norwegian setting, internal incentives to work systematically on all organizational levels to improve quality and safety became stronger as a result from the introduction of the Quality Improvement Regulation. It also paved way for a stronger system perspective to regulatory inspectors’ assessment of the services [40, 46, 73]. This increase in attention to performance overall is partly justified by previous international findings demonstrating scarce impact on quality and safety from external inspection of compliance with standards [5, 6]. The attention also reflects a broader development in regulatory culture in general, where implementing “self-regulatory approaches” is considered part of the solution in ensuring relevance and ownership [73]. Thus, external inspection aiming at ensuring a certain level of performance may be assumed to have greater relevance and realism to the healthcare organizations’ internal improvement processes [4, 74, 75]. Depending on the way external inspections are conducted and if and how they engage and involve staff, have been demonstrated in the literature to be critical to how the findings from external inspection are perceived and followed up internally in the hospitals [9].

Mirrored by regulatory theory, the success of regulation depends on the responsiveness of the regulators to the regulatees [76–79]. Responsive regulation as a dynamic theoretical construct, considers persuasion of the regulatees and/or internal capacity building the primary option for regulators- before more punitive actions are tried [78, 79]. The public perception of the model as being fair and legitimate is however a key precondition for the model to work as intended [78, 79]. The findings in our study displayed variation in the inspectors’ responsiveness, depending on the case complexity, severity of the case and availability of internal resources. Different inspection approaches were applied, with different level of encouragement to stakeholder involvement. The most frequently applied approach was self-assessment, whilst onsite inspections were infrequent. The two approaches illustrate the two “extremes” of available external inspection approaches. On one hand, self-assessment is conducted and led by the hospital itself and to that extent it represents a way of ensuring direct stakeholder involvement from the regulatee undergoing inspection (i.e., the hospital). Onsite inspection on the other hand is an approach where the external inspection bodies oversee the process, a “full-scale version” of external inspection, where the regulatee undergoing inspection is approached from the “outside”. It seems crucial, based on the findings, that information and communication flows both ways, between internal and external stakeholders, regardless of the approach(es) of response chosen by the regulatory inspection body. The adversarial principle, meaning that a party should be allowed to comment on any submission from its opponent, is part of why responsiveness and tight communication should be considered valuable [80]. Studies in the past [9–11, 81, 82] seem to not have directly touched upon this argument, but our study implicates more awareness to this specific liberal democratic right held by the patients and the informal caregivers. An increase in the sensitivity of the inspectors towards the adversarial principle would perhaps also underpin what some of our participants emphasized: that the formal objective of external inspection did not always meet the level of accountability and responsibility claimed from the perspectives of the patient and/or informal caregivers. Past research has shown how using a restorative approach in responses to serious incidents in healthcare may counter the affected patients, informal caregivers, hospital organizations and health professionals’ sense of epistemic injustice [83, 84]. The right to have one’s voice heard could to some degree explain why it is important to include all sorts of implicated stakeholders in the aftermath of a serious incident. Based on our findings, patients and/or informal caregivers were not always seen as constructive in their effort to enlighten the case complexity. Not seeing these stakeholders as valid sources of knowledge, regardless of their approach to the process, should not be a standalone argument for omitting them from the external inspection process. Studies have in fact indicated that information from next of kin resulted in changes in the regulatory inspectors’ conclusions [25].

The framework of “value driven regulation” provides further interesting input to these matters, stressing how the “societal value” of inspection may increase due to the inspectors’ collection and interpretation of the regulatees’ activities [85]. To this study’s understanding, the mere fact that patients and/or informal caregivers become informed and are allowed to provide information to the inspectors themselves is imperative for the legitimacy of external inspection as a governmental, regulatory activity. The key question according to “value driven regulation” however, is to answer *who* (e.g. who are the actors having informal power) should be doing *what* (e.g. how are quality standards defined) to achieve *which* (e.g. what is the objective of regulation) societal value [85]. Indeed, our participants indicated that the scope of external inspection struggled with the balance between externally formulated objectives and the level of public expectation to what external inspection productively could contribute to. This was related to different perceptions of quality and safety, for instance, implying that inspectors and patients interpreted the societal value; regulatory objective differently. From a theoretical point of view, these tensions also relate to how reconciliation by epistemic and restorative justice may facilitate a more unified cross-level understanding of what purpose regulation should serve, and to whom it should serve its purpose [86, 87]. Considering that there often is a gap between work as imagine (regulations and policies) and work as imagine (work practices as these unfold in reality) it seems sensible for any government that seeks to align formalities and legitimacy to be more responsive to different perceptions of societal values [76, 78, 79].

Playing into the challenges of stakeholder interaction in external inspection is also the aspect of power distribution. Our findings provide indications of a gap between the internal work practices of the inspectors against external expectations, similar to other studies [30, 34, 81]. The power distribution between regulatory inspectors on one hand and patients and informal caregivers on the other is by nature uneven. Although a comprehensive set of patient rights exists, only regulatory bodies have the power to make decisions and potentially put sanctions into effect [13, 15, 16]. The hospitals are situated in the middle of this power dynamic, having to deal with both inspectors, patients, informal caregivers as well as having internal obligations to protect its employees [40–43]. It is not irrelevant to think that this power dynamic has an impact on interaction and communication, which may hamper the ability and willingness to agree on the narrative and accept outcomes from the decision-making processes. Findings from a Dutch study confirmed that perspectives provided by patients or informal caregivers were downplayed by inspectors if these contradicted the perspectives from health professionals involved [34]. The power distribution thus unfortunately reinforces the tensions between inspectors, hospital organizations and patients and informal caregivers.

Another paradox is the recent report of further governmental requirements and expectations to hospital internal follow up of serious incidents and an increase in attention to stakeholder involvement in these internally based processes, in parallel with less attention to the subsequent increase in workload and lack of competence [58, 88]. This was reflected in our participants’ responses.

### Implications of the current system design - comprehensive pressures on the regulatory inspectors

Unlike our findings, previous studies have explored how bodies of external inspection could encourage hospitals to prepare for onsite inspection by self-assessing clinical practice and involving clinicians prior to the onsite inspection visit [10]. Others have investigated the impact of an increasingly layered system of governance and regulation of healthcare quality, showing how inspectors aimed at gaining control of quality of care in parallel with dependency on professional self-regulation by the healthcare organization undergoing inspection, introducing challenges of having to deal with a variety of stakeholders and regulatory instruments [89]. Comparing the Norwegian system of regulatory inspection to structures in other countries shows how the Dutch system shares similar multiple layers in its regulatory design and external evaluation processes [4, 18]. However, in contrast to Norway, the Dutch system combines accreditation standards and processes of external inspection, implying that it is possible to design the regulatory system in ways that ensure organizational autonomy of the hospital by the application of inspection, in parallel with structured compliance by the means of accreditation [4]. Based on existing literature, it is not possible to assess which one of the system designs that poses more, or less, pressure on the regulatory inspectors. Our findings however indicate issues with the current Norwegian system design of multiple system levels involved in inspection processes. Balancing internal stakeholders (the NBHS versus the County Governor level) and various external stakeholders were described as conflicted by our participants.

**Fig. 1:**
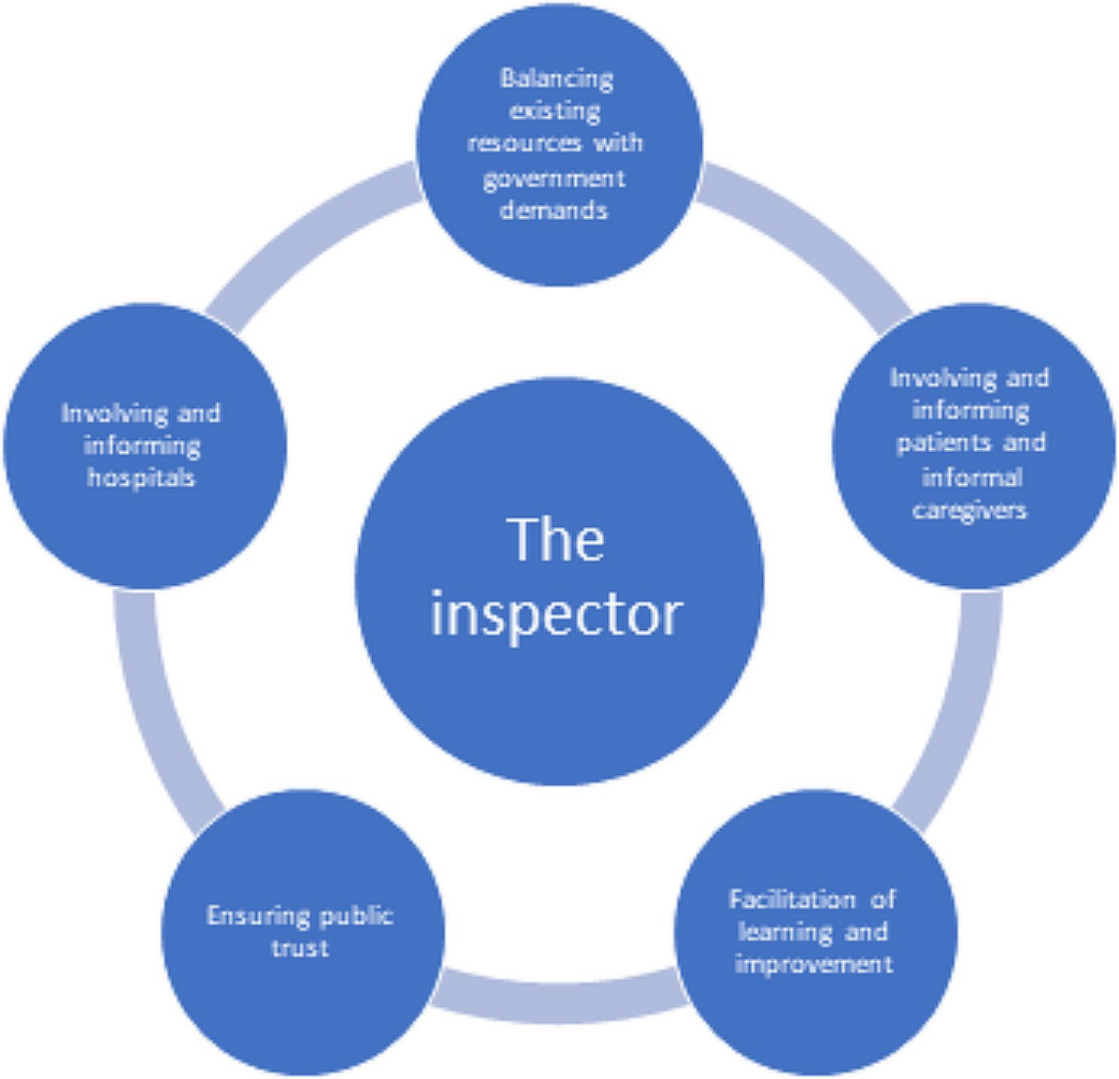
Pressures on the regulatory inspectors in the incident-based inspection process

Although external inspection as external assessment of quality and safety has its overall objective of contributing to system-level improvement, and previous research has shown external inspection as moving into more system-oriented approaches [25], our study indicates that the different expectations and trade-offs required in external inspection processes makes it difficult to meet the needs of all stakeholders involved. The trade-offs are similarly reflected in responsive regulation theory: even if regulators choose to approach the regulatees in accordance with restorative justice principles, it may also fail because “noncompliance is neither about a lack of goodwill to comply nor about rational calculation to cheat. It is about management not having the competence to comply” (79 p.119). The complexity of the serious incident leaves the team of inspectors to balance different governmental expectations with the expectations from the patient and informal caregivers, as well as considering the perspectives of the hospital and health professionals. This results in comprehensive pressure on the inspectors, illustrated in Fig. 1 below.

The findings and discussions demonstrated in this paper, display how the NBHS and County Governors decide between different approaches of responding to an incident, how they assess and process serious incidents with constant trade-offs. Part of the trade-off is *who* they choose to involve and *how* they choose to involve hospitals, healthcare professionals, patients, and informal caregivers. Implications from this study could serve as insight into a complex landscape of external inspection. We suggest that government bodies should acknowledge the trade-offs in external inspection, and more openly discuss the implications of the existing system design to ensure meaningful and relevant stakeholder involvement.

### Strengths and limitations of the study

This study’s main strength is the novel focus on the links between approaches of responding in external inspection and involvement strategies of stakeholders such as hospitals and healthcare professionals, patients and/or informal caregivers. The application of mixed methods represents a rigorous exploration of the perspectives of regulatory external inspectors (interview data) and macro level expectations (document data). The study sample could have been larger; however, this was supplemented by the inclusion of documentary data. The included documents and interviews were in Norwegian. Thus, translation of the interview transcripts or document quotes into English during the analytical process may not have captured all the linguistic nuances. Although the study’s findings are limited to a specific structural, and cultural context, the qualitative mixed methods approach contributes to a fuller understanding of a highly relevant topic regardless of cultural context: regulatory inspections of hospitals after serious incidents. Further studies are required to extend findings related to how the wider system of accountability structures may support the internal work practices in the regulatory system of external inspection, to better align its formal objectives with the public expectations. As the findings reported in this paper are limited to the experiences and perspectives of *inspectors*, future research could benefit from including experiences and perspectives of *patients and informal caregivers*.

## Conclusion

The study found that inspectors are formally expected to apply five different approaches in their responses to serious incidents and ensure stakeholder involvement in the following external inspection. The study provides new insight into how strategies for stakeholder involvement in external inspection entails complex and challenging interaction with stakeholders due to different views, resource demands and lacking competence. The inspectors considered balancing the formal objectives with the expectations of the public, hospitals, patients, and informal caregivers, to be a challenging part of their job as inspectors. Cross level interaction is suggested as a possible way forward and may contribute to positively informing the inspectors’ decision-making processes and case complexity, and remedy some of the uneven power distribution between the stakeholders. Based on our findings, and with support from previous literature, our study thus suggests that the regulatory system of external inspection and its available approaches of responding to serious incidents in the Norwegian setting is currently not designed to accommodate the complexity of needs from stakeholders at the levels of hospital organizations, patients, and informal caregivers altogether.

### Electronic supplementary material

Below is the link to the electronic supplementary material.

Supplementary Material 1

## Data Availability

Data may be obtained from a third party and are not publicly available. Data retrieved from the interviews are not publicly available due to the risk of identification but may be available from the corresponding author upon reasonable request and with permission from the participant(s).

## Declarations

### Provenance and peer review

Not commissioned; external peer review.

## List of abbreviations

MHCS: Ministry of Health and Care Services
NBHS: Norwegian Board of Health Supervision
