# Supplementary material for: External inspection approaches and involvement of stakeholders’ views in inspection following serious incidents - a qualitative mixed methods study from the perspectives of regulatory inspectors
Source: BMC Health Serv Res. 2024 Mar 6;24:300. doi: 10.1186/s12913-024-10714-9 (PMC10919011; doi:10.1186/s12913-024-10714-9)
Supplement: Supplementary file 1 — Supplementary Material 1 [file 12913_2024_10714_MOESM1_ESM.pdf]

Interview Guide - English language version, original version in Norwegian

Applied to participants situated at the Norwegian Board of Health Supervision (NBHS) or the County Governors. Hence, “external inspection body” refers to both governmental bodies.

The terms “case” and “incident” are used interchangeably throughout the interview guide.

The interviewer starts by introducing herself, providing details about the study aim, the timeline of the study, how the data from the interview is being processed, anonymized, stored and applied in the study, resulting in scientific article publication(s).

Questions

- Please introduce yourself by age, professional background and experience, and current role and position (except your name).
- Would you please describe your role in external inspection following serious incidents?
- Would you please describe the procedural process following notification or transfer of an incident?
- How do the external inspection bodies determine which incidents to process further?
- In cases where the initial phase indicates inadequate care and unsound professional practice, how is the information assessed and how does the external inspection body proceed to follow-up the case?
- What types of cases become delegated or transferred to the County Governors?
- How does the external inspection body involve patients or informal caregivers during the assessment phase of the case, following the serious incident?
- How does the external inspection body involve health professionals who have directly been involved in the serious incident, during the assessment phase of the case?
- In what ways does involvement of patients, informal caregivers and health professionals show in the reports produced by the external inspection body?
- How does the external inspection body consider the value of the hospitals’ self-assessment?
- What sorts of feedback have you received from the hospitals regarding the self-assessment template?
- What sorts of competences do you consider valuable for the hospitals do possess, for the self-assessment approach to be useful for learning purposes?
- Who did contribute to the development of the self- assessment template? Did you play a part in the process? Who else did (e.g. clinicans, lawyers)?

- What are the pros and cons of the application of self-assessment with respect to involvement?
- What are the foundational theoretical concepts of risk and safety in the external inspection body's assessment of serious incidents?
- What are the existing supporting governmental documents concerning strategies of involvement in external inspection?
- What are your thoughts about the construction of team members in external inspection (e.g. gender balance, interdisciplinarity)?
- Does team member construction affect involvement strategies?
- Could you please tell us about your experiences related to application of onsite inspections?
- Which elements are brought into the assessment of whether to do onsite inspection?
- Could you give us any examples of serious incident where onsite inspection was considered and determined?
- Have you experienced cases where onsite inspection was considered sensible, but did not occur?
- Could you give any examples of a serious incident where the external inspection body did not find onsite inspection sensible, but where the patient and/or informal caregivers requested onsite inspection?
- We are aware that the external inspection bodies have attracted criticism for conducting few onsite inspections. What do you think about this criticism?
- In your contact with the hospitals – the first meeting- who do you normally communicate with? And how do you communicate?
- What are your experiences of involving and communicating with hospital quality and safety departments?
- What are your experiences of involving and communicating directly with patients and/or informal caregivers?
- What do you think are the benefits of involving patients and/or informal caregivers? Any disadvantages?
- What about involving health professionals? What are the benefits and disadvantages in that regard?
- In what scenarios or cases would it be inappropriate to involve patients and/or informal caregivers?
- Have you ever experienced any unpleasant encounters with any of the stakeholders involved, following a serious incident?
- The external inspection bodies have received media scrutiny - has it affected your work? If yes, in what ways?
- Has (the media scrutiny) affected your motivation and confidence in your work? If yes, how has is affected your motivation and confidence?
- How do you consider media reports to influence public trust in external inspection following serious incidents (i.e. media reports published in VG; the largest newspaper in Norway)? Have these media reports resulted in any specific changes (in your work practices)?
- Based on your experience, how would you describe an ideally designed external inspection? What would you consider to be sensible and useful external inspection?
- The COVID 19 pandemic: did it play into the degree/extent of involvement in external inspection?

- Could you please describe any features of development to external inspection approaches, following serious incidents?
- Have you experienced any changes in the level of expectations to stakeholder involvement in external inspection?
- How do you consider the current practices regarding risk-based external inspection?
- What are your experiences related to cases where the hospital involved, and the external inspection body have divergent perceptions of the case?
- Have you experienced any unrealistic expectations or demands in your work?
- How do you consider the balance between the power of external inspection controlling compliance with standards for sound professional practice and the trust in the hospitals' ability in doing self-assessment?
- What do you think should be the role of external inspection bodies following serious incidents?

We have asked you the questions we planned on asking, however if you would like to add anything, please feel free to share.
